# Clinical Significance and Anatomical Considerations of Apical Patency in Endodontic Therapy: A Comprehensive Review

**DOI:** 10.3390/dj14050294

**Published:** 2026-05-13

**Authors:** Hidetaka Ishizaki, Takashi Matsuura

**Affiliations:** 1 Department of Periodontology and Endodontology, Nagasaki University Graduate School of Biomedical Sciences, Nagasaki 852-8588, Japan; matsuurat@nagasaki-u.ac.jp; 2 Okaguchi Dental Clinic, Tokyo 102-0083, Japan

**Keywords:** apical patency, root canal preparation, postoperative pain, cone-beam computed tomography, NiTi instruments

## Abstract

**Background:** The primary goal of root canal treatment is the prevention and healing of apical periodontitis through the meticulous elimination of pathogenic bacteria and infected tissues. Within this framework, apical patency remains a fundamental yet debated clinical concept. **Objectives:** This review aims to evaluate the clinical significance of maintaining apical patency, its influence on postoperative discomfort, and the technical strategies required for predictable negotiation. **Methods:** We performed a comprehensive review of existing literature, including clinical studies and recent meta-analyses, focusing on the correlation between patency maneuvers and postoperative pain, the role of preoperative CBCT imaging, and the efficacy of specialized negotiation instruments and motor kinematics. While patency facilitates thorough debridement, evidence regarding its impact on postoperative pain is conflicting, with recent meta-analyses suggesting it may actually alleviate discomfort intensity. Preoperative CBCT was identified as essential for identifying complex anatomy, such as the MB2 canal. Furthermore, the use of specialized files and reciprocating motor modes enhances the predictability of glide path establishment. **Conclusions:** Although failure to achieve patency does not always dictate a negative outcome, it is associated with improved long-term healing. Clinicians should prioritize “Anatomical Patency”—respecting original morphology—over forceful “Operative Patency” to ensure procedural integrity and clinical success.

## 1. Introduction

The primary goal of root canal treatment is to promote healing and prevent apical periodontitis by eliminating pathogenic bacteria, necrotic pulp tissue, and infected dentin from the root canal system [1]. Although most people agree on this aim, the clinical process of creating and keeping apical patency, which is the topic of the proposed review, is not only a matter of technical difficulty but also clinical controversy. While clinicians typically strive for apical patency to establish the canal and working length, the procedure is often challenging. Apical patency due to inappropriate instrumentation can lead to procedural accidents such as canal transportation, instrument separation, or the creation of ledges, zips, and perforations. Furthermore, maintaining patency may inadvertently trigger postoperative pain, leading to conflicting clinical perspectives on its necessity.

Despite numerous studies on the technical aspects of canal negotiation, a significant gap remains in differentiating between successful apical penetration and the preservation of original anatomy. Much of the existing literature fails to distinguish between negotiation that respects the natural canal path and negotiation that achieves the apex at the cost of iatrogenic alteration. This review intends to critically synthesize the evidence regarding the clinical impact of apical patency, particularly its debated relationship with postoperative pain and long-term healing. Furthermore, it aims to introduce a new clinically relevant framework by defining the distinction between “Anatomical Patency”—a technique that respects and preserves the original root canal morphology—and “Operative Patency”, which prioritizes apical penetration, often at the expense of anatomical integrity. By assessing contemporary diagnostic tools and specialized instrumentation strategies, this paper provides a comprehensive guide for clinicians to predictably achieve anatomical patency while minimizing iatrogenic risks and enhancing treatment outcomes.

A structured search was performed to identify peer-reviewed articles focusing on apical patency. The search was conducted in PubMed and the Cochrane Library for studies published up to late 2025. Keywords included “apical patency”, “root canal preparation”, “postoperative pain”, and “cone-beam computed tomography”. Articles focusing on non-human studies or those with limited clinical relevance were excluded.

## 2. Apical Patency

Patency is defined as the ability of a small file to pass through the apical foramen without resistance and is a critical concept in endodontic therapy [2]. Achieving apical patency enables the comprehensive removal of apical pathogenic bacteria, necrotic pulp tissue, and infected dentin and establishes a precise working length for canal preparation.

Since Kakehashi et al. laid the foundation for our understanding of the link between bacteria and apical periodontitis in 1965 [3], the primary goal of endodontic therapy has been to reduce the microbial load below the threshold of the host’s immune response to promote healing, as highlighted by Siqueira JF Jr [4]. Furthermore, Song et al. identified missed canals, leaky seals, and underfilling as primary causes of non-surgical root canal failure [5], suggesting that persistent intraradicular infection remains the central challenge. The inherent complexity of the root canal system—characterized not only by curvatures and calcifications but also by isthmuses, fins, undercuts, and lateral canals—underscores the extreme difficulty in achieving complete bacterial eradication [6,7,8,9,10,11,12,13,14,15,16]. Consequently, achieving apical patency to facilitate thorough chemomechanical debridement—followed by precise, three-dimensional obturation to the apex—remains a cornerstone of predictable endodontic success [17]. However, negotiating and shaping severely curved or calcified canals presents significant clinical challenges. Iatrogenic complications occurring during this process, such as canal transportation, ledge formation, apical zipping, and perforations, are critical factors that directly compromise treatment outcomes [18,19,20,21,22,23,24,25,26].

Eleftheriadis et al. reported canal curvature as the single most critical factor contributing to perforations [27]. Supporting this, Haji-Hassani et al. reported that apical perforation remains a frequent endodontic mishap across both arches and regardless of tooth type [28]. These findings suggest that many perforations result from iatrogenic errors—such as ledges or zips—when the clinician fails to negotiate the original canal path during attempts to restore patency. Furthermore, Gorni and Gagliani demonstrated that an “altered” root canal morphology resulting from transportation or perforation significantly reduces the success rate of retreatment [21]. This underscores the paramount importance of achieving patency while strictly adhering to the original canal anatomy during initial treatment.

Maintaining apical patency is a critical step in endodontics, as it ensures both thorough chemomechanical debridement of the apex and a void-free obturation. However, patency is often compromised when dentinal debris becomes packed in the apical third during instrumentation. Such blockages hinder effective enlargement and irrigation, potentially leading to iatrogenic errors, including ledge formation, transportation, or perforation [19,29]. To address this, Buchanan LS advocated a technique known as “apical patency”, which involves passing a small hand K-file slightly beyond the apical foramen between successive instrument changes. This practice prevents the accumulation of debris and avoids the need for canal transportation. Moreover, it preserves the original canal morphology and facilitates the effective flow of irrigants to the critical apical region [30].

## 3. Apical Patency and Postoperative Pain

While maintaining apical patency is expected to improve the healing of apical periodontitis and potentially reduce postoperative discomfort, concerns persist that the procedure may exacerbate initial symptoms. Some studies suggest that patency maneuvers can increase the incidence of short-term postoperative pain. For instance, Iftikhar Akbar et al. reported that although the overall prevalence of postoperative pain did not differ significantly between groups, the mean pain scores during the first 5 days were significantly higher in the patency group. Furthermore, patients in the patency group required analgesics at a higher rate [31]. They suggested that maintaining patency in cases of necrotic pulp may actually increase the likelihood of postoperative pain [31]. In their study, root canals were instrumented with Ni-Ti files (ProTaper Universal), irrigated with 3% NaOCl and 17% EDTA, and medicated with calcium hydroxide. Since these were necrotic cases, several factors may have contributed to the pain: the extrusion of infected debris or irrigants beyond the apical foramen, or increased internal pressure from placing hydraulic temporary cement over a flowable intracanal medicament, which could have forced the calcium hydroxide apically.

Shubham et al. also observed significantly higher postoperative pain in the patency group, noting that these levels were further influenced by the preoperative pulpal status and initial pain levels [32]. Shubham et al. reported that the patency-maintained group showed higher mean pain scores regardless of the number of visits [32]. While no difference was found based on pulp status in single-visit treatments, they noted that in multi-visit treatments, the vital pulp group experienced higher postoperative pain than the non-vital group. Although irritation from irrigation or obturation is possible, it is also notable that stainless-steel files were used for instrumentation; these are less efficient at removing debris, which may lead to the extrusion of contaminated chips during maintenance of patency. Similarly, Gagan Chaudhari demonstrated that patients in the patency group had significantly higher pain scores at 6 and 24 h than those in the non-patency group. Although this difference diminished by 48 h and was completely resolved within seven days, the study concluded that maintaining apical patency is indeed associated with more intense discomfort during the early postoperative phase [33]. They treated irreversible pulpitis in a single visit using Ni-Ti files, 5.25% sodium hypochlorite, and gutta-percha obturation with AH Plus sealer [33]. However, the specific irrigation protocol was not described, and the obturation method was only listed as “AH Plus and gutta-percha”. This leaves open the possibility that the irrigant acted as an irritant or, if vertical compaction was used, that the sealer or gutta-percha may have extruded slightly. While postoperative pain is likely influenced by a complex combination of factors, these findings underscore that clinicians should always prioritize a “gentle touch” and meticulous technique during endodontic procedures.

In contrast, Arias et al. observed that maintaining apical patency in non-vital teeth was associated with significantly lower levels of postoperative pain. They noted that factors such as preoperative pain and mandibular tooth location tended to prolong any discomfort that did occur [34]. Similarly, Arora et al. reported that patency maneuvers had no significant impact on postoperative pain in mandibular molars presenting with pulpal necrosis and apical periodontitis [35]. More recent systematic reviews and meta-analyses provide further insight into this topic. A 2018 meta-analysis by Abdulrab et al. concluded that maintaining apical patency was not associated with increased postoperative pain [36]. Furthermore, a 2024 meta-analysis by Xiqian et al. found that—although the quality of the evidence ranged from low to moderate—maintaining patency significantly reduced the intensity and incidence of postoperative discomfort [37]. These meta-analyses, including References [34,35], conclude that maintaining apical patency does not increase postoperative pain.

Conflicting perspectives have been reported in previous studies regarding the relationship between maintaining apical patency and postoperative pain. While some studies have reported that maintaining patency significantly increases early postoperative pain, recent meta-analyses suggest that it may alleviate pain intensity and reduce the incidence of discomfort. This discrepancy in findings is likely not due to a single variable but rather to complex interactions among multiple clinical and technical confounding factors.

First, the initial status of apical periodontitis significantly influences the prognosis of postoperative pain. In cases presenting with severe preoperative spontaneous pain or acute symptoms, mechanical stimulation from patency maneuvers may exacerbate apical inflammation, thereby increasing the risk of heightened pain. In contrast, in cases of chronic lesions, establishing patency and associated apical decompression may alleviate discomfort.

Furthermore, incidental irritation during treatment procedures is a critical confounding factor. In multi-visit treatments involving intracanal medicaments such as calcium hydroxide, unintended pressure during patency maneuvers may force the medicament beyond the apical foramen. This is particularly prevalent when stainless-steel files are used, as their lower efficiency at removing debris makes the extrusion of contaminated chips more likely. It is speculated that the chemical and mechanical irritation resulting from such “procedural errors” may artificially inflate postoperative pain scores.

These conflicting clinical results regarding apical patency are likely not a reflection of the technique’s validity, but rather a result of the clinician’s varying ability to control critical variables—such as inflammatory status, irrigant dynamics, and instrumentation—with the core issue being that this may lead to the incidental extrusion of infected debris or intracanal medicaments. Any substance forced into the periapical tissue, whether in vital or necrotic cases, inevitably triggers an adverse inflammatory reaction, such as pain or swelling. Previous reports suggesting that patency increases pain likely involved cases where procedural accidents occurred due to insufficient debris removal or inadequate clinical control. In contrast, when patency is achieved with a “gentle touch” that respects the original canal morphology, it facilitates thorough debridement and reduces the microbial load, thereby potentially alleviating discomfort. Consequently, clinical education should shift from a focus on mere “Operative Patency” (forceful penetration) to the standardization of “Anatomical Patency,” which prioritizes preventing iatrogenic extrusion while ensuring procedural integrity. By respecting the original root canal morphology and employing gentle approaches to achieve apical cleaning, clinicians can minimize postoperative pain while consistently achieving successful healing outcomes.

## 4. Strategies for Achieving Patency

The difficulty of achieving patency varies significantly depending on the tooth type and the unique anatomy of each canal. Root canal systems often exhibit complex configurations, including calcified constrictions, severe curvatures, and internal variations such as branching or merging of canals. Before attempting patency, essential preparatory steps include meticulous radiographic interpretation, adequate access-cavity preparation, and removal of cervical constraints (coronal flaring). To predictably achieve Anatomical Patency—negotiation that respects the original canal morphology—a systematic approach starting from the coronal aspect is essential. The following strategies, from radiographic assessment to coronal pre-flaring, are framed not just as routine procedures but as the foundational steps required to prevent iatrogenic alteration of the canal path.

### 4.1. Radiographic Assessment

**Figure 1:**
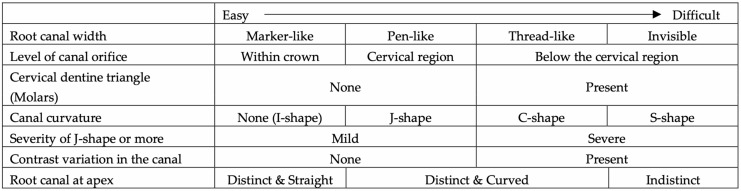
Key parameters for preoperative radiographic interpretation and their impact on the level of difficulty in achieving apical patency.

Preoperative intraoral radiographs are indispensable for analyzing canal morphology. A sudden change in radiographic density or a sharp shift in the canal outline often suggests anatomical complexities, such as branching, merging, or abrupt curvature [38]. Factors such as root canal width, the prominence of “endodontic triangles”, and the degree of curvature collectively dictate the level of difficulty. These diagnostic parameters must be thoroughly evaluated preoperatively to anticipate potential challenges during negotiation (Figure 1).

Iatrogenic complications, such as canal transportation, ledge formation, perforation, and instrument separation, are primary concerns in endodontic therapy that frequently lead to treatment failure. Regarding the impact of separated instruments on clinical outcomes, McGuigan et al. noted that, although drawing a definitive conclusion remains challenging, the presence of preoperative apical periodontitis significantly reduces the likelihood of successful healing [39]. These complications are largely attributed to errors in interpreting the initial canal anatomy, inadequate access cavity preparation, or technical lapses during the negotiation and shaping phases. Therefore, accurate anatomical assessment before intervention is paramount. In this regard, AAE 2015/2016 Updated Position Statement [40] advocates for the strategic use of preoperative Cone-Beam Computed Tomography (CBCT) in initial treatment, stating in Recommendation 3 that:

“Limited FOV CBCT should be considered the imaging modality of choice for initial treatment of teeth with the potential for extra canals and suspected complex morphology, such as mandibular anterior teeth, and maxillary and mandibular premolars and molars, and dental anomalies.”

**Figure 2:**
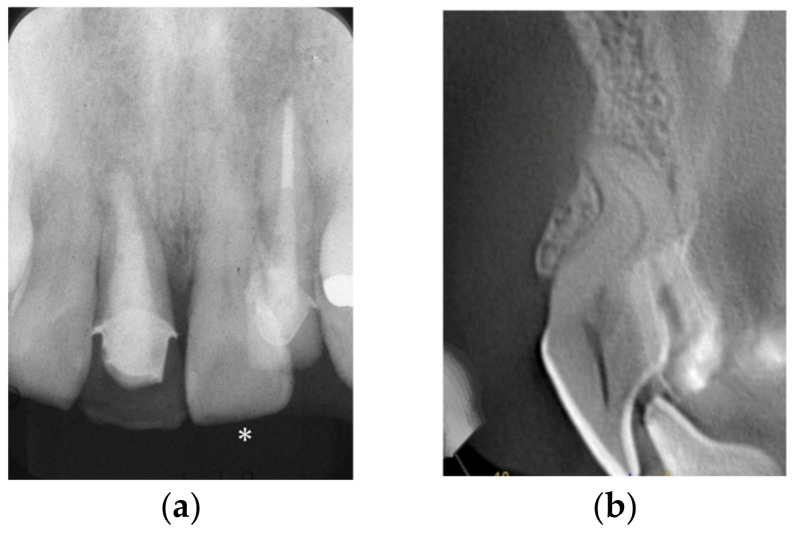
Comparison of diagnostic imaging for a maxillary left central incisor (∗). Periapical radiograph (**a**) and corresponding preoperative CBCT scans (**b**). The three-dimensional imaging reveals the intricate internal anatomy and provides critical information for establishing a safe path to the apex.

Although clinicians are familiar with the standardized root canal anatomy of each tooth type [41], the frequent occurrence of anatomical variations and extra canals necessitates a more cautious approach. In cases where complex morphology is suspected, preoperative CBCT is highly recommended to gain a three-dimensional understanding of the canal number and configuration (Figure 2).

**Figure 3:**
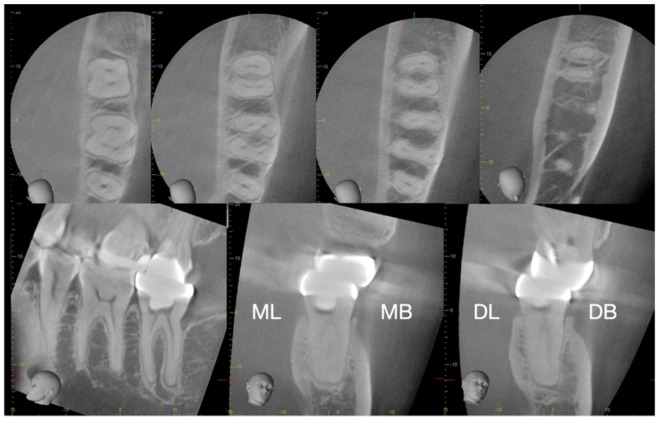
CBCT analysis of a mandibular left second molar. The cross-sectional images highlight how the canal orifices are obscured by endodontic triangles in both the mesiodistal and buccolingual dimensions. Such findings underscore the necessity of three-dimensional imaging to recognize constraints that would otherwise be invisible on standard periapical radiographs.

While conventional two-dimensional intraoral radiographs allow for the assessment of mesiodistal cervical constraints (endodontic triangles), they fail to visualize interferences in the buccolingual dimension. This limitation is particularly critical in the mesial roots of mandibular molars, which frequently house two canals. In such cases, one must be vigilant for endodontic triangles that restrict access not only mesiodistally but also buccolingually (Figure 3). Identifying these three-dimensional constraints is essential to ensure an unobstructed path for the file to the apical third.

**Figure 4:**
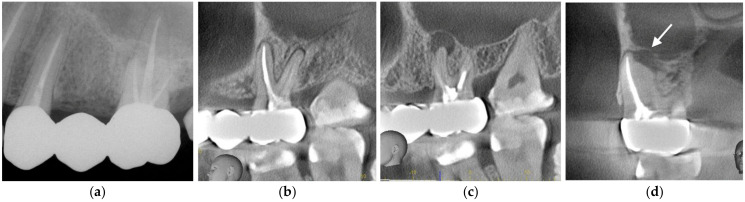
Periapical periodontitis associated with a missed MB2 canal in a maxillary left first molar. (**a**–**d**) Despite the initial appearance of adequate obturation in the primary canals, the 3D imaging reveals a secondary infection originating from the untreated MB2 canal. These figures illustrate the diagnostic value of CBCT in identifying elusive anatomy.

Karabucak et al. reported that the second mesiobuccal (MB2) canal of maxillary molars is frequently overlooked; notably, the presence of an untreated canal is associated with a 4.38-fold increase in the prevalence of periapical lesions compared with cases in which all canals are treated [42]. In clinical practice, a case may appear to have all three primary canals successfully obturated to the apex on a standard two-dimensional radiograph; however, an undetected MB2 can harbor persistent infection, leading to periapical pathology and symptomatic distress (Figure 4a–d). This underscores the fact that meticulous preoperative interpretation of radiographs and CBCT scans is paramount for preventing missed anatomy and, ultimately, avoiding endodontic failure.

### 4.2. Access Cavity Preparation, Orifice Enlargement, and Straight-Line Access

Following the initial radiographic interpretation, the focus shifts to establishing a proper access cavity and identifying the canal orifices. Depending on the orientation and dimension of the canals, the orifices and the coronal third of the root canal system must be carefully enlarged and refined. In recent years, the clinical validity of the minimally invasive access cavity (MIAC) has gained significant attention in terms of tooth structure preservation [43,44]. Silva et al. categorized these modern access designs into several types, including Traditional AC, Conservative AC, Ultra-conservative AC, Truss AC, Caries-driven AC, and Restoration-driven AC [45]. However, while these constricted designs may favor biomechanical strength, they are often associated with critical clinical compromises. Reports indicate that excessive conservation can lead to missed canals, inadequate chemomechanical debridement, compromised obturation quality, and an increased risk of instrument separation [44,46,47,48,49]. Al-Helou et al. advocate for traditional access cavity designs over minimally invasive approaches to ensure treatment predictability [50]. Similarly, Dina Abdellatif et al. emphasize that access morphology should not be predetermined; rather, it should be tailored to the unique clinical requirements of each case [51]. Corsentino G et al. reported that there was no significant difference in fracture resistance when the number of remaining walls was the same; however, even with the same access cavity design, fracture resistance was significantly higher with four remaining walls than with three or two walls, indicating that the number of remaining walls is more critical to fracture resistance than the access cavity design [52]. For this reason, while the preservation of tooth structure is a fundamental goal, it must never be prioritized at the expense of procedural integrity or inadequate disinfection.

**Figure 5:**
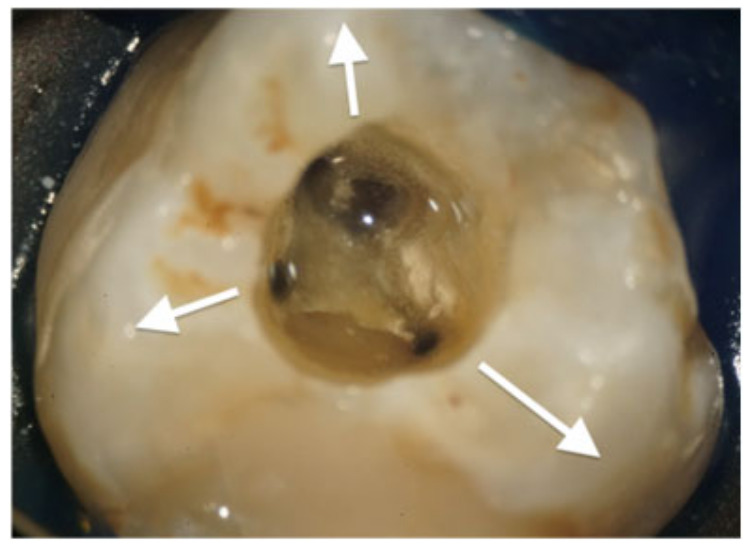
Following orifice identification, the coronal third is enlarged to establish straight-line access. Straight line access is achieved by expanding outwards.

Within this context, establishing “straight-line access” remains a cornerstone of endodontic therapy. This involves the selective removal of cervical dentin interference to allow the initial filing to enter the coronal portion of the canal without deflection. Ensuring an unobstructed path to the apical third is essential to minimize instrument stress and facilitate more controlled negotiation of the canal anatomy [53]. Traditionally, rotary instruments such as Gates-Glidden drills or Peeso reamers have been employed for this purpose. More recently, dedicated nickel–titanium (NiTi) orifice openers have become widely utilized. When using these instruments, it is crucial to perform enlargement with an outward focus to ensure safety and efficiency (Figure 5).

**Figure 6:**
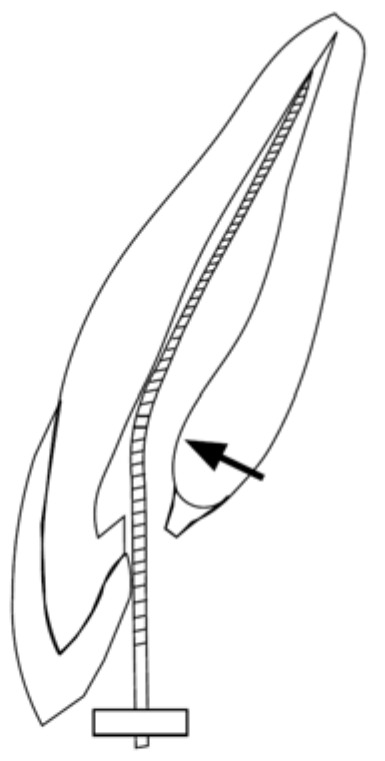
The lingual shoulder (Black arrow)—a prominent shelf of dentin in the cervical region of anterior teeth—restricts the insertion angle of the file. Proper removal of this interference is necessary to facilitate unhindered apical negotiation.

In maxillary and mandibular anterior teeth, where access is typically gained through the lingual or palatal surface, the “lingual shoulder” at the cervical region often restricts the path of the file. Removing this lingual shoulder is essential to eliminate file deflection and achieve true straight-line access to the apical region (Figure 6).

Establishing straight-line access is not merely a preliminary step for canal visibility; it is the fundamental clinical safeguard for Anatomical Patency. By removing cervical interferences, the clinician minimizes file deflection in the apical third, thereby ensuring that the instrument follows the natural canal path rather than creating iatrogenic deviations like ledges or transportations.

While modern MIAC aims for maximal preservation of tooth structure, it must be balanced against the biological mandate of predictable disinfection. From the perspective of ‘Endodontic Biology,’ the primary objective is to minimize cumulative irritation to the organism by reducing the microbial load to a level that is manageable by the host immune system. Over-conservative access that compromises canal negotiation or irrigation efficacy can result in persistent infection, necessitating repeated interventions that pose greater cumulative biological risks than a slightly more extensive initial access cavity.

### 4.3. Coronal Pre-Flaring

Coronal pre-flaring refers to the initial enlargement of the canal orifice to eliminate constraints in the cervical third of the root canal system. This step offers several clinical advantages. Pecora et al. noted that relying solely on a clinician’s tactile sense to determine canal length can be inaccurate; however, pre-flaring the cervical and middle thirds significantly improves the precision of measuring the anatomical diameter at the working length [54]. Similarly, Tennert et al. reported that cervical pre-flaring enhances the accuracy of apical size determination [55]. Regarding apical negotiation, pre-flaring eliminates coronal interferences, facilitating the smoother introduction of both hand and rotary files. This reduction in frictional resistance allows the instrument to reach the apex more predictably. Coronal pre-flaring serves as a critical prerequisite for achieving Anatomical Patency. Eliminating coronal constraints reduces the frictional resistance (stress) on the file tip as it navigates the complex apical anatomy. This stress reduction allows for a more delicate ‘scouting’ of the root canal, ensuring that the original morphology is preserved while the apical foramen is predictably negotiated. Ultimately, the success of root canal therapy and apical patency depends heavily on the proper management of the canal orifice and the coronal portion of the canal.

## 5. Specialized Files for Apical Negotiation

In addition to standard reamers and K-files, several hand instruments have been specifically designed to facilitate apical negotiation. The characteristics of these specialized files are discussed below.

### 5.1. D-Finder (Mani, Inc., Japan)

The D-Finder is a stainless-steel instrument with a unique D-shaped cross-section and a single cutting edge on a 0.02 taper. A significant portion of its circumference serves as a large radial land, allowing the file to “glide” through the canal rather than aggressively cut dentin. This design feature effectively minimizes the risk of procedural accidents such as instrument engagement (binding), ledge formation, or perforation. While standard hand files are typically used with reaming or filing motions, the D-Finder is engineered with low cutting efficiency specifically to support a “push-type” advancement through the canal. This specialized longitudinal movement reduces torsional stress, thereby decreasing the risk of file separation from torsional fatigue. The D-Finder is available in sizes #8, #10, #12, and #15.

### 5.2. Glide-Finder (Mani, Japan)

The Glide-Finder (GF) is a stainless-steel instrument designed for both canal wall instrumentation and negotiation. It features a specialized 3% taper in the apical 3.5 mm to provide high buckling resistance, while the remainder of the shaft maintains a standard 2% taper. For the technically minded clinician, its primary advantage lies in its specialized geometry: the cross-sectional shape transitions from a square at the tip to a rectangular configuration toward the handle. This unique shift in cross-sectional design yields a stiff, resilient apical portion for effective penetration while maintaining a flexible coronal shaft for better navigation. The GF is particularly effective for negotiating and establishing a glide path in calcified canals where active cutting of the canal walls is required. It is available in sizes #8, #10, #12, and #15.

### 5.3. C+ File (Dentsply Maillefer, Switzerland)

The C+ File is a stainless-steel instrument with a square cross-section, similar to a standard K-file. However, it features a 4–5% taper in the apical 4 mm, which significantly enhances its buckling resistance and reduces tip deformation. Beyond the apical 4 mm, the taper decreases to 1%. Compared to conventional K-files, the C+ File offers superior resistance to vertical pressure and is less prone to bending, allowing for more efficient force transmission to the tip. This makes it highly effective for negotiating constricted or calcified canals using a standard filing motion. It is available in sizes #6, #8, #10, and #15.

### 5.4. C-Pilot File (VDW, Germany)

The C-Pilot File is a stainless-steel K-file with a constant 2% taper that undergoes a specialized heat treatment to increase the instrument’s overall stiffness. Designed specifically for orifice searching and negotiation, it has a rigidity equivalent to that of a file two sizes larger. A unique feature of the C-Pilot is its radiopaque markings on the shaft, which facilitate the confirmation of working length during radiographic assessment. The lineup includes sizes #6, #8, #10, #12.5, and #15.

Each of the aforementioned files possesses distinct mechanical characteristics. The initial step involves evaluating root canal morphology via intraoral radiography to select the appropriate instrument. For highly calcified or curved canals, C+ Files, C-Pilot Files, or G-Files (GF) are utilized to achieve patency while simultaneously enlarging the canal.

However, because the C+ File is designed with a sturdier tip and a higher taper to enhance penetration, forced application in curved canals may result in ledging or perforation. In such anatomical cases, a transition to C-Pilot Files or GF is recommended. The D-File (DF) is characterized by reduced cutting efficiency, which prevents “screwing-in” effects and allows for safe scouting of the canal; it is best suited for canals with mild-to-moderate stenosis. Since the DF is not intended for significant dentin removal, it is advisable to follow its use with GF or similar instruments to enlarge further and secure the patency. Notably, both C+ and C-Pilot Files are available in ISO size #06, making them the instruments of choice when navigating extremely constricted canals.

## 6. Mechanical Negotiation and Instrumentation

Traditionally, apical negotiation and canal enlargement have been performed using manual stainless-steel files. However, a landmark study by Walia et al. in 1988 demonstrated that NiTi hand files possessed two to three times the elastic flexibility and superior resistance to torsional fracture compared to conventional stainless-steel K-files [56]. This pivotal research highlighted the clinical efficacy of NiTi instruments, particularly in managing severely curved canals. Subsequent integration of NiTi technology into endodontic practice has enabled clinicians to achieve efficient canal shaping while more predictably preserving the original root canal anatomy [57,58,59,60]. Despite these advantages, a significant concern remains: unlike stainless-steel files, which often exhibit visible deformation before failure, NiTi instruments are prone to sudden fracture without warning [57,61]. To ensure safe instrumentation with NiTi files, the establishment of a “glide path”—defined as a smooth radicular tunnel from the root canal orifice to its terminus—is highly recommended before canal enlargement [62]. In this context, Berutti et al. demonstrated that in an S-shaped root canal model, a “mechanical glide path” created with NiTi rotary instruments (e.g., PathFile, Dentsply Sirona) was more effective at preserving the original canal anatomy compared to a “manual glide path” established with traditional K-files [63]. Consequently, the clinical protocol of establishing a glide path—using either NiTi rotary instruments or manual K-files—before full-scale canal shaping has become a widely accepted standard. Nevertheless, for the initial negotiation of the canal and the precise determination of working length, manual stainless-steel K-files remain the instrument of choice. Their superior tactile feedback and physical properties ensure a safer approach when navigating the apical third for the first time, minimizing the risk of procedural errors before transitioning to automated systems.

Protocols are now emerging that employ rotary files rather than manual K-files for this initial negotiation. For instance, the “ProTaper Ultimate” system (Dentsply Sirona) promotes a “Rotary-first” concept; according to internal manufacturer testing, approximately 63% of canals could be successfully negotiated and shaped starting with the “Slider” instrument, bypassing manual pre-flaring. It has been reported that NiTi instruments enable consistent canal shaping regardless of the clinician’s experience [60]. If a “NiTi-only” workflow, from negotiation to enlargement, were universally predictable, it would undeniably offer significant benefits to the patient. However, achieving patency in canals where preoperative intraoral assessments indicate excessive calcification, severe curvatures (including apical curves), or complex branching remains a formidable challenge. Once a ledge or transportation is iatrogenically created, the original canal anatomy is permanently altered. Therefore, despite the allure of automated systems, initial scouting and negotiation conducted meticulously with manual files is recommended to allow the clinician to negotiate the canal while maintaining critical tactile feedback and to ensure the safest possible trajectory for high-difficulty cases presenting with these anatomical challenges.

Furthermore, significant innovations have been made not only in NiTi metallurgy but also in the kinematics of endodontic motors. To mitigate the risk of file separation, reciprocating motion was developed as an alternative to continuous rotation [64]. Another example is the Optimum Glide Path 2 mode integrated into the Tri Auto ZX2 (Morita), which utilizes a complex sequence of alternating clockwise and counterclockwise movements. Despite these sophisticated drive modes and automatic functions designed to reach the apex safely, clinicians must remain cautious about the biological risks. Kılıç et al. demonstrated that even with such advanced technology, the risk of apical debris extrusion persists [65]. This indicates that technological precision does not eliminate the potential for periapical irritation. Consequently, while these innovations enhance procedural predictability, they should not replace a meticulous and gentle technique. Avoiding the extrusion of dentinal debris and irrigants remains a fundamental priority to minimize postoperative discomfort and ensure a favorable biological response, regardless of the equipment employed.

As NiTi instruments, endodontic motors, and their sophisticated kinematics continue to evolve, we are entering an era of unprecedented precision. It is expected that in the near future, these further advancements will enable even less-experienced clinicians to achieve apical negotiation, glide path establishment, and canal shaping with ease while faithfully preserving the original root canal anatomy.

## 7. Apical Patency and Treatment Outcomes

The primary objective of endodontic therapy is the elimination of pathogenic bacteria to facilitate healing and prevent apical periodontitis; mechanical debridement through apical patency is a critical component of this process. However, achieving patency is not always feasible in all clinical scenarios. Machado et al. reported that while patency was achieved in 93% of non-vital teeth with apical lesions, the success rate for negotiation dropped to 85.4% in vital cases, highlighting the inherent anatomical challenges [66].

Whether the presence or absence of apical patency definitively influences the long-term success of initial or retreatment cases remains a subject of debate. Healing often occurs even when patency is not established. Machado concluded that there is insufficient scientific evidence to link patency directly to the success rate of root canal therapy in necrotic cases with apical periodontitis [67]. Similarly, Allen (2012) reported that maintaining apical patency did not significantly affect endodontic outcomes [68]. While achieving patency is clinically desirable, it is not an absolute prerequisite for success. The reduction in the intracanal microbial load to a level that the host’s immune system can manage—achieved through chemical disinfection with sodium hypochlorite and intracanal medicaments such as calcium hydroxide—remains the fundamental driver for the healing of apical periodontitis.

Conversely, a 2024 systematic review by Kuzhanchiathan et al. reported that maintaining apical patency nearly doubled long-term success rates in both initial and nonsurgical retreatment cases. Their findings suggest a high probability that patency significantly enhances postoperative healing [69]. Achieving patency while maintaining the original anatomical morphology may improve success rates compared with cases in which patency is not achieved. However, clinician-induced patency involving apical perforation may leave untreated areas in the apical region and thus cannot be considered an improvement in the success rate. It is crucial to distinguish between these two and ensure that patency is achieved while preserving the anatomical integrity. Whether apical patency is a definitive predictor of long-term success remains a focal point of endodontic research, and further high-quality prospective studies are eagerly anticipated to clarify its clinical impact.

## 8. Conclusions

While failure to achieve apical patency does not inevitably dictate a negative outcome, clinicians should strive to negotiate the full canal length whenever possible to ensure maximum intracanal disinfection. However, forceful negotiation that results in canal transportation, ledge formation, or apical perforation should be critically termed “Operative Patency.” This is a result-oriented practice that risks iatrogenic diversion of the canal from its natural course, achieving apical penetration at the expense of anatomical integrity. The inherent stiffness, aggressive cutting ability, and high buckling resistance of traditional specialized negotiation files may inadvertently contribute to this type of iatrogenic trauma. Ultimately, our clinical goal should transcend mere penetration; we must keep “Anatomical Patency” in mind as a technique that respects and preserves the original root canal morphology throughout the negotiation process. Future research needs to develop and validate techniques and technologies to maintain these conditions of anatomical Patency, and clinical education should be based on this qualitative difference rather than on the quantitative achievement of apical penetration.

## Data Availability

No new data were created or analyzed in this study.

## Abbreviations

The following abbreviations are used in this manuscript:

AAE	American Association of Endodontists
CBCT	Cone-Beam Computed Tomography
NiTi	Nickel–Titanium
MB2	Second Mesiobuccal Canal
